# Indents

**Published:** 1911-11-15

**Authors:** 


### INDENTS.

**☞Brief Articles of Technical or Scientific Value will receive notice and comment. Contributions invited.**

*Death Due to Poisoning by Infected Tooth.* It was learned to-day that the sudden death of Dr. Charles E. Wood, staff physician of the Brooklyn Rapid Transit Company was due to blood poisoning, resulting from an infected tooth. About a week ago Dr. Wood complained of sharp pains in one of his teeth, but no apprehension was felt that it would have serious consequences. Treatment by a dentist was not deemed necessary. Very rapidly, however, the infection of the ulcer spread throughout the mouth until it became necessary to call into consultation Dr. John Jennings, of 164 Halsey street. Blood poisoning being immediately suspected, the surgeon ordered Dr. Wood removed to the Brooklyn Hospital. At the institution it was found that the infection had spread so rapidly that the entire mouth and lips of the sufferer were affected. Everything that was known to medical science to allay the infection was done, and several consultations were held, but to no avail. During the few days the patient lingered at the hospital it was necessary to feed him through a silver tube in the throat. Dr. Wood suffered intense pain. On Wednesday death finally came. A short interview with a member of the household contradicted the report that the infection of the tooth had been due to careless work on the part of some dentist, as it was stated that the deceased had not consulted any dentist when the trouble first started.—*Brooklyn* (N. Y.) *Eagle*, February 3, 1911.

*Model Separation.* I find that by coating the impression with a saturated solution of paraffin dissolved in gasoline (first staining the impression by means of aqueous solution of carmine, or marking with indelible pencil while plaster is still moist) I can accomplish the same result in about three minutes, for the impression will be ready for pouring as soon as the plaster can be mixed after the paraffin solution is applied, and it will be unnecessary for the impression to be soaked, for, soak as much as you will, no air bubbles will escape from it, and a good model is secured. Another point in favor of the paraffin solution is that the coating is so thin that there is no possible chance of any disparity between size of impression and model, as there might be should the varnishes used happen to be a little too thich, and the annoyance of having the caps on the bottles forever stuck fast just as one wants to use the contents of the bottle in a hurry, is overcome.

The ease with which the impression and model may be separated may be illustrated by the fact that I have so loosened model from impression while tapping handle of impression tray to loosen it from impression, that model could be separated by slight movement with the hands. This has happened with cases where all teeth were present in mouth, and impression had been taken for correcting irregularities.—Dr. Kilbourne in *Summary*.

